# Repurposing Penfluridol to combat mycobacteria via bioenergetic collapse

**DOI:** 10.1128/spectrum.01919-26

**Published:** 2026-08-18

**Authors:** Yunbing Li, Xiaofan Li, Guangfen Zhang, Chunli Li, Changting Yin, Yuanyuan Li, Jiani Jiang, Yi Yang, Zhiqiang Yang, Yinli Liu, Xiangkun Zeng, Jiayi You, Yi Liu, Ahui Li, Chang Jiang, Chen Xu, Ning Dong

**Affiliations:** 1 Department of Emergency Medicine, Second Affiliated Hospital, Zhejiang University School of Medicine 26441, Hangzhou, China; 2 Department of Medical Microbiology, Experimental Center, Suzhou Medical College of Soochow University 74565, Suzhou, China; 3 Key Laboratory of Chemical Biology and Molecular Engineering of Ministry of Education, Biomedical and Health Laboratory in Shanxi Province, Institutes of Biomedical Sciences, School of Life Science, Shanxi University 692800, Taiyuan, China; 4 Biomedicine and Health Laboratory in Shanxi Province, Taiyuan, China; 5 Department of Pathology, Children’s Hospital, Zhejiang University School of Medicine, National Children’s Regional Medical Center, National Clinical Research Center for Child Health 26441, Hangzhou, China; 6 Department of Laboratory Medicine, School of Medicine, Jiangsu University 191611, Zhenjiang, China; Second Affiliated Hospital of Soochow University, Suzhou, China

**Keywords:** Penfluridol, tuberculosis, mycobacteria, PMF

## Abstract

Drug-resistant *Mycobacterium tuberculosis* (Mtb) infections constitute a persistent and severe public health crisis worldwide. Standard anti-tuberculosis (anti-TB) therapies often fail to eradicate resistant strains, resulting in poor clinical outcomes and underscoring the demand for new treatment options. Drug repurposing offers a practical and time-efficient approach to accelerate the development of new anti-mycobacterial regimens. In this work, we evaluated the antimycobacterial potency of penfluridol (PL), a Food and Drug Administration (FDA)-approved antipsychotic drug, and explored its functional mechanism against mycobacteria. Our experimental data revealed that PL compromises mycobacterial membrane integrity and depletes the proton motive force (PMF), leading to disrupted energy metabolism and mycobacterial growth inhibition. PMF depletion disables energy-reliant efflux systems, facilitates intracellular PL accumulation, and further deteriorates membrane damage, creating a sustained bactericidal effect. Notably, continuous PL exposure rarely induces stable drug resistance. These observations suggest that PL can be repurposed for treating drug-resistant TB and confirm that targeting bacterial bioenergetics is an effective strategy to overcome clinical antimicrobial resistance.

**IMPORTANCE:**

Tuberculosis (TB) remains a major threat to global public health, and the widespread emergence of drug-resistant strains has substantially compromised the efficacy of existing anti-TB regimens. The scarcity of approved anti-TB drugs, combined with lengthy treatment regimens and frequent therapeutic failure, further exacerbates the global TB crisis, highlighting an urgent need for safe and effective alternative therapies. Drug repurposing serves as a time-saving and reliable strategy to accelerate anti-TB drug development. This study explores the antimycobacterial efficacy and unique working mechanism of the clinically approved agent penfluridol (PL). Our findings identify a feasible candidate for treating drug-resistant TB and provide a valuable bioenergetic-targeting strategy to combat bacterial antimicrobial resistance.

## OBSERVATION

Tuberculosis (TB) caused by *Mycobacterium tuberculosis* (Mtb) remains a leading cause of infectious disease mortality. According to 2022 World Health Organization (WHO) data, approximately 10 million new active TB cases and 1.6 million TB-related deaths occurred worldwide annually (1), highlighting a major global health threat.

The emergence of multidrug-resistant (MDR) and extensively drug-resistant (XDR) Mtb strains has severely compromised the efficacy of standard anti-TB therapy, leading to increased treatment failure and mortality (2). Bacterial resistance is mediated not only by genetic mutations but also by cell wall, remodeling metabolic reprogramming, and epigenetic modulation, which collectively facilitate immune evasion and therapeutic failure (3, 4). Collectively, these mechanisms contribute to treatment failure, prolonged therapy, and the further transmission of drug-resistant strains.

Long treatment courses of 6–9 months for drug-susceptible TB and up to 2 years for drug-resistant TB frequently result in poor treatment adherence, therapeutic failure, and disease recurrence (5, 6). To date, approximately 10 anti-TB drugs have been approved for clinical use. Notably, only three new drugs have emerged in the past two decades, including bedaquiline (FDA 2012), pretomanid (FDA 2019), and delamanid (EMA 2014, not FDA-approved) (7). Limited drug availability further exacerbates the crisis, creating an urgent need for safer, shorter-course, and more effective anti-TB therapies.

**Fig 1:**
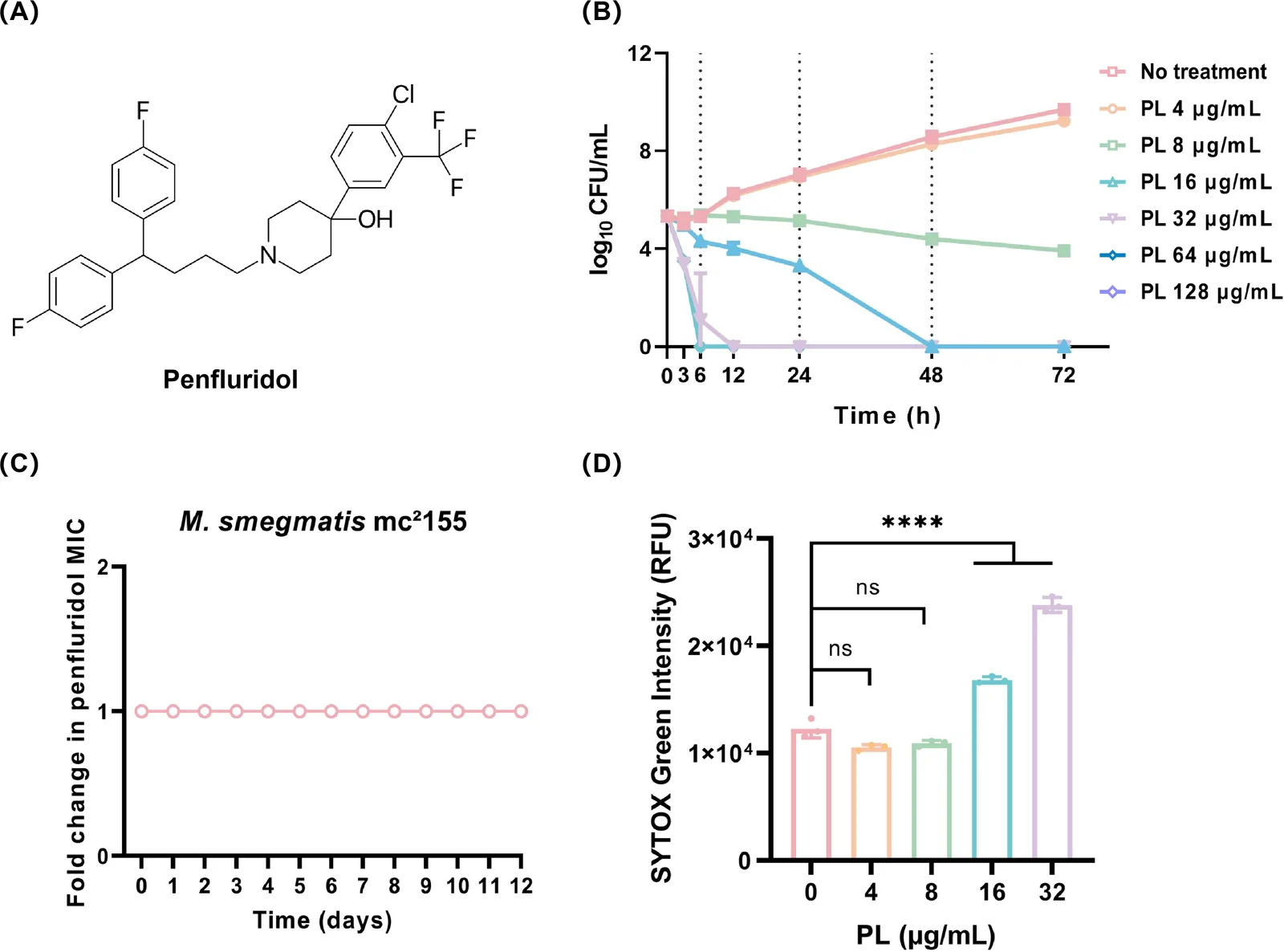
Inhibitory effects of PL on *M. smegmatis* mc² 155. (**A**) Chemical structure of PL. (**B**) Time-killing assay of *M. smegmatis* mc² 155 subjected to treatment with PL. (**C**) The development of PL resistance in *M. smegmatis* mc² 155 was evaluated via 12-day serial passage with sub-inhibitory PL concentrations 0.5, 1, 2, 4, and 8 μg/mL. PL treatment effectively inhibited the *in vitro* emergence and progression of *M. smegmatis* resistance. (**D**) The influence of PL on cell membrane permeability of *M. smegmatis* mc² 155 was determined by using the SYTOX dye. Statistical analysis was evaluated by unpaired two-tailed Student’s *t*-test. Data represent mean ± SEM from three independent biological experiments. *P < 0.05, **P < 0.01, ***P < 0.001, and ****P < 0.0001. NS, not significant.

Drug repurposing represents a time-efficient strategy to expand the therapeutic arsenal. We, therefore, screened a library of FDA-approved drugs for antimycobacterial activity and identified penfluridol (PL), an antipsychotic agent (8), as a promising hit. Its lipophilic structure (Fig. 1A) suggested potential interference with mycobacterial membrane integrity and energy metabolism, and its well-established clinical safety profile prompted us to pursue further mechanistic characterization.

In our preliminary study, we evaluated the *in vitro* activity of PL against *M. smegmatis* mc² 155, a fast-growing and non-pathogenic mycobacterium widely used as a model organism in mycobacterial research. The broth microdilution assay following the Clinical and Laboratory Standards Institute (CLSI) guidelines determined the minimum inhibitory concentration (MIC) of PL against *M. smegmatis* mc² 155 as 16 μg/mL. Partial inhibition of bacterial growth was observed at concentrations as low as 4 μg/mL (1/4 MIC), while complete killing (no detectable CFU on agar plates) was achieved at 16 μg/mL by 48 h, at 32 μg/mL by 24 h, and at 64–128 μg/mL within 6 h (Fig. 1B). A 12-day serial passage assay was performed to evaluate the risk of resistance development (9). Throughout the 12-day passaging period, the MIC of PL remained strictly at its initial value of 16 μg/mL, and no resistant mutants were recovered (Fig. 1C). Based on the time-kill profiles in Fig. 1B, we selected 16 μg/mL (1 × MIC) and 32 μg/mL (2 × MIC) for mechanistic assays to compare the onset and amplification of membrane damage and PMF dissipation. Wider concentration ranges were included for dose–response assays.

The mycobacterial cell membrane is critical for intracellular survival and intrinsic drug resistance, rendering it an attractive therapeutic target (10). Given its essential role, we hypothesized that PL exerts its antibacterial activity by disrupting membrane integrity. SYTOX Green staining was used to evaluate membrane permeability. This fluorescent nucleic acid stain is impermeant to live cells with intact membranes. However, upon membrane damage, it penetrates the cells and binds to nucleic acids, emitting intense green fluorescence. Our results demonstrated that PL treatment resulted in a significant, dose-dependent increase in fluorescence intensity, indicating severe membrane permeabilization that contributes to bacterial cell death (Fig. 1D).

**Fig 2:**
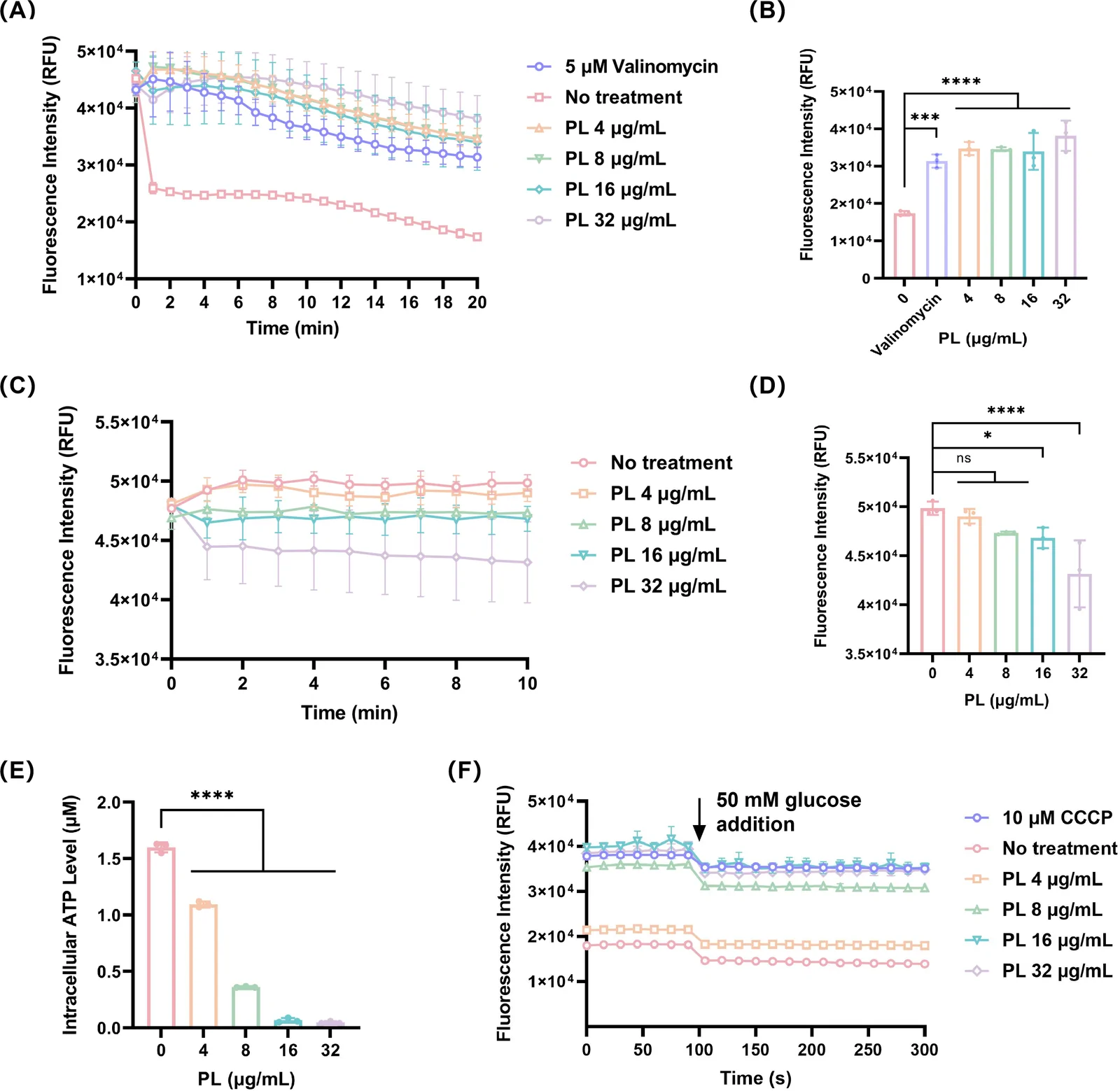
Effects of PL on *M. smegmatis* mc^2^ 155. (**A**) ΔΨ of *M. smegmatis* mc^2^ 155 treated with PL was measured using DiSC_3_(5) (excitation/emission: 622/670 nm). Valinomycin, a K^+^ ionophore, served as the positive control. (**B**) Fluorescence intensity of DiSC_3_ (5) at 20 min. (**C**) ΔpH in BCECF-AM-labeled cells exposed to PL. (**D**) Fluorescence intensity of BCECF-AM (excitation/emission: 488/535 nm) at 10 min. (**E**) Quantification of intracellular ATP levels in *M. smegmatis* mc^2^ 155 after PL treatment. Raw luminescence signals were converted to absolute ATP concentrations (μM) using an ATP standard curve. (**F**) Efflux pump activity in PL-treated *M. smegmatis* mc^2^ 155 assessed by Nile Red staining. Fluorescence intensity was monitored for 100 s, followed by addition of 50 mM glucose, and recording was continued for another 200 s. Statistical analysis was evaluated by unpaired two-tailed Student’s *t*-test. Data represent mean ± SEM from three independent biological experiments. *P < 0.05, **P < 0.01, ***P < 0.001, and ****P < 0.0001. NS, not significant.

Furthermore, we investigated the effect of PL on the proton motive force (PMF), a critical driver of bacterial ATP synthesis composed of membrane potential (ΔΨ) and pH gradient (ΔpH) (11). Disruption of PMF components impairs bacterial energy metabolism and triggers bactericidal effects in gram-positive bacteria, which, in turn, induces membrane permeabilization and cell death (12, 13). We, therefore, hypothesized that PL exerts its bactericidal activity by dissipating PMF. To test this, we monitored using the potential-sensitive dye 3,3′-dipropylthiadicarbocyanine iodide (DiSC₃(5)). PL treatment induced a potent, dose-dependent increase in fluorescence, indicating rapid ΔΨ dissipation. Notably, at high concentrations (32–64 μg/mL), PL exhibited an effect comparable to valinomycin, a classic positive control and well-established membrane potential disruptor (Fig. 2A and B). Given that ΔΨ depolarization often triggers a compensatory increase in ΔpH to maintain homeostasis, we further determined intracellular pH using BCECF-AM. Although a slight compensatory elevation in ΔpH was observed, it was insufficient to counteract the massive loss of ΔΨ, leading to a collapse of total PMF (Fig. 2C and D). As the PMF serves as the driving force for ATP synthesis (14), we next quantified intracellular ATP levels. Consistent with PMF collapse, intracellular ATP levels decreased significantly following PL exposure (Fig. 2E), confirming that PL disrupts bacterial bioenergetics.

Since PMF and ATP serve as essential energy sources for bacterial efflux pumps, a Nile Red accumulation assay was applied to evaluate efflux activity (15). Nile Red, a substrate for multiple bacterial efflux pumps, exhibits intense fluorescence upon incorporation into the bacterial membrane but is rapidly extruded by functional pumps. PL treatment increased intracellular Nile Red fluorescence in a dose-dependent manner, with concentrations of 16–32 μg/mL producing effects comparable to the classic PMF uncoupler CCCP, indicating significant suppression of PMF-dependent efflux (Fig. 2F). Notably, glucose supplementation (marked by the arrow) failed to restore efflux activity in both CCCP- and PL-treated groups, indicating that PL induces sustained PMF disruption rather than transient energy depletion. Impaired efflux prevents PL extrusion and promotes intracellular drug accumulation, which further exacerbates PMF dissipation and membrane damage, thereby forming a positive feedback loop that amplifies the bactericidal activity. These data support a stepwise model. PL first damages the membrane, leading to PMF collapse. PMF loss reduces ATP production and disables efflux pumps, allowing PL to accumulate inside the cell. This accumulation worsens membrane injury and PMF dissipation, creating a self-reinforcing cycle that drives sustained bactericidal activity.

PL disrupts the mycobacterial membrane and dissipates PMF, which is essential for bacterial energy synthesis, membrane stability, and efflux activity, making it an attractive antimicrobial target for both Gram-positive and Gram-negative bacteria (13). However, most PMF-targeted agents have notable limitations. Classic uncouplers (CCCP, FCCP, and nigericin) cause severe host cytotoxicity (16). Clinically available PMF-impairing anti-TB drugs, such as bedaquiline, delamanid, and clofazimine, frequently induce QTc prolongation and are susceptible to acquired or cross-resistance. The first-line anti-TB drug pyrazinamide also collapses mycobacterial PMF under acidic conditions, whereas its clinical application is restricted by obvious side effects. In addition, rifampicin and isoniazid exhibit narrow therapeutic windows and a high prevalence of clinical drug resistance (17–19).

Apart from suppressing gram-positive pathogens, such as *Staphylococcus aureus*, and removing biofilms, PL enhances the bactericidal efficacy of colistin against drug-resistant, gram-negative bacteria by increasing outer membrane permeability (20–22). Nevertheless, previous studies only reported membrane morphological alterations without verifying whether PMF dissipation mediates the bactericidal effects of PL against different bacterial strains. As an FDA-approved drug with a clear safety profile, PL exhibits unique advantages for anti-TB drug repurposing.

In conclusion, PL exhibits potent bactericidal activity against *M. smegmatis* with a low risk of resistance development. Nevertheless, this study remains limited to *in vitro* bacterial experiments based on non-virulent *M. smegmatis*, which cannot fully reflect the pharmacological characteristics of PL against pathogenic Mtb. Further studies are, therefore, necessary to validate PL activity against Mtb strains and clinical drug-resistant Mtb isolates, evaluate its efficacy inside mycobacteria-infected macrophages, and assess its *in vivo* efficacy and safety using animal models. Collectively, our findings highlight the therapeutic potential of targeting bacterial bioenergetics and support the further development of PL as a promising repurposed candidate for combating drug-resistant TB.

## Data Availability

All data generated or analyzed during this study are included in this article and its Supplemental materials and methods.

## SUPPLEMENTAL MATERIAL

Supplemental materials and methods spectrum.01919-26-s0001.docx Supplemental materials and methods

ASM does not own the copyrights to Supplemental Material that may be linked to, or accessed through, an article. The authors have granted ASM a non-exclusive, world-wide license to publish the Supplemental Material files. Please contact the corresponding author directly for reuse.
