# Supplementary material for: Repurposing Penfluridol to combat mycobacteria via bioenergetic collapse
Source: Microbiol Spectr. 2026 Aug 18;14(10):e01919-26. doi: 10.1128/spectrum.01919-26 (PMC13689427; doi:10.1128/spectrum.01919-26)
Supplement: Supplementary file 1 [file spectrum.01919-26-s0001.docx]

**Supplementary Materials**

**Materials and Methods**

**Bacterial strains and reagents**

*Mycobacterium smegmatis* mc^2^ 155 (ATCC 700084) strain was cultured in Middlebrook 7H9 (Himedia) medium. Penfluridol (PL)（P160772, Aladdin）was initially dissolved in dimethyl sulfoxide (DMSO).

**Antimicrobial susceptibility assay**

The minimum inhibitory concentrations (MICs) of PL were determined with microbroth dilution assay (1). The results were interpreted following the Clinical & Laboratory Standards Institute (CLSI) M24-A2 guideline (2).

**Determination of the bactericidal effect of PL**

The overnight culture of strain mc^2^ 155 was initially diluted 1:100 in fresh 7H9 broth and cultured to logarithmic phase (OD_600_ = 0.4–0.6). Subsequently, different concentrations of PL were introduced to the cultures under standard incubation conditions at 37 °C. Bacterial viability was assessed at specified time intervals 0, 3, 6, 12, 24, 48, 72 h using established quantification methods with biological replicates. Killing curves were drawn by plotting log10 CFU mL^−1^ against time (h) using GraphPad Prism 9.0.

**Resistance development assay**

The development of PL resistance was evaluated according to the previously reported methods (3). Briefly, sequential culture of mc^2^ 155 was performed with sub‑inhibitory levels of PL to obtain resistant mutants. The serial passage was sustained over a period of 12 days.

**Detection of membrane permeability**

Membrane permeability was tested using SYTOX Green. Briefly, the isolate was cultured to OD_600_=0.5, treated with different concentrations of PL for 2 h, then centrifuged, washed once with normal saline, and resuspended to OD_600_=0.2. SYTOX Green was added to a final concentration of 1 µM, and the isolate was incubated at room temperature for 10 min in the dark. Fluorescence intensity was measured using a microplate reader (excitation: 488 ± 10 nm; emission: 523 ± 10 nm) (4).

**Membrane potential assay via DiSC_3_(5) fluorometric assay**

*M. smegmatis* mc^2^ 155 was inoculated in 7H9 broth and cultured until an OD_600_ of 0.5 was reached. Bacterial cells were harvested by centrifugation, washed once with PBS, resuspended, and adjusted to an OD_600_ of 0.2. KCl and DiSC₃(5) were added to final concentrations of 100 mM and 1 μM, respectively, followed by incubation at room temperature for 5 min in the dark. The stained bacterial suspension was added to a black 96-well plate. PL at different final concentrations (0-64 μg/mL) was added , while the positive control group received valinomycin (1 μM). Fluorescence intensity was monitored continuously for 20 min using a microplate reader with an excitation wavelength of 610 nm and an emission wavelength of 660 nm (5).

**ΔpH Measurement**
A fresh single colony of *M. smegmatis* mc² 155 was inoculated into 7H9 broth and cultured to OD₆₀₀=0.5. After washing three times with PBS and resuspending, 10 μM BCECF-AM fluorescent dye (Beyotime) was added for 30 min in the dark. Stained bacteria were transferred to a 96-well plate, initial fluorescence intensity was measured, and then PL at different concentrations was added. Fluorescence intensity was continuously monitored for 10 min using a microplate reader with excitation/emission parameters set at 488 nm and 535 nm, respectively (6).

**Quantification of intracellular ATP**
The intracellular ATP concentration of mc^2^ 155 was quantified using an ATP detection Kit (Beyotime, China*)*. Bacteria was inoculated into 7H9 broth and cultured at 37 ℃, 220 rpm to OD_600_ = 0.5, then resuspended in PBS. Bacteria were treated with PL at final concentrations of 0-64 μg/mL for 3 h, and the OD_600_ was recalibrated to 0.5. Bacterial cells were lysed with ATP assay lysis buffer and lysozyme. After centrifugation at 12,000 g at 4 ℃ for 5 min, the supernatant was collected (6). ATP assay solution was added to a 96-well black plate, incubated at room temperature for 5 min, then mixed with the collected supernatant. Luminescence intensity was detected using a microplate reader, with relative RLU values reflecting intracellular ATP concentration.

**Assessment of efflux activity**

*M. smegmatis* mc^2^ 155 was cultured to an optical density at an OD_600_ of 1.0 and resuspended in PBS supplemented with 1 mM MgCl₂. After standing for 15 min, 10 μM CCCP was added, and the mixture was incubated at room temperature for 15 min. MS was then treated with PL at different final concentrations (0-64 μg/mL), with the positive control group receiving an additional 10 μM CCCP, followed by incubation at room temperature for 15 min. Subsequently, 5 μM Nile Red was added, and the samples were incubated with shaking for 1 h. After standing at room temperature for 1 h, the bacterial cells were centrifuged, resuspended in PBS containing the same concentrations of the aforementioned agents, and immediately subjected to fluorescence intensity detection using a microplate reader (excitation wavelength: 552 nm; emission wavelength: 636 nm). Fluorescence intensity was continuously measured for 100 s, after which 50 mM glucose was added, and the monitoring was continued for an additional 200 s (7).

**Statistical analysis**

All data were performed with at least three biological replicates and shown as means ± SD. An unpaired *t*-test between two groups was performed to calculate the *P* value among different groups. GraphPad Prism 9.0 was used to calculate the *P* values (* *P* < 0.05, ** *P* < 0.01, *** *P* < 0.001, **** *P* <0.0001).

**REFERENCES**

1.	Huang L, Li Y, Xu C, Zhou M, Wang T, Wang T, Wang J, Tang J, Li Y, Dong N. 2023. A novel virulence plasmid encoding yersiniabactin, salmochelin, and RmpADC from hypervirulent Klebsiella pneumoniae of distinct genetic backgrounds. Antimicrob Agents Chemother 67:e0093523.

2.	Woods GL, Brown-Elliott BA, Conville PS, Desmond EP, Hall GS, Lin G, Pfyffer GE, Ridderhof JC, Siddiqi SH, Wallace Jr RJ. 2011. Susceptibility testing of mycobacteria, nocardiae, and other aerobic actinomycetes.

3.	Dokrungkoon T, Tulyaprawat O, Suwannakarn K, Ngamskulrungroj P. 2023. modeling of isoniazid resistance mechanisms in

H37Rv. Frontiers in Microbiology 14.

4.	Kim W, Zhu WP, Hendricks GL, Van Tyne D, Steele AD, Keohane CE, Fricke N, Conery AL, Shen S, Pan W, Lee K, Rajamuthiah R, Fuchs BB, Vlahovska PM, Wuest WM, Gilmore MS, Gao HJ, Ausubel FM, Mylonakis E. 2018. A new class of synthetic retinoid antibiotics effective against bacterial persisters. Nature 556:103–+.

5.	Xie MM, Chen KC, Chan EWC, Chen S. 2022. Synergistic Antimicrobial Effect of Colistin in Combination with Econazole against Multidrug-Resistant Acinetobacter baumannii and Its Persisters. Microbiology Spectrum 10.

6.	Cai JJ, Shi JR, Chen C, He MP, Wang ZQ, Liu Y. 2023. Structural-Activity Relationship-Inspired the Discovery of Saturated Fatty Acids as Novel Colistin Enhancers. Advanced Science 10.

7.	Wang MM, Chan EWC, Wan YK, Wong MHY, Chen S. 2021. Active maintenance of proton motive force mediates starvation-induced bacterial antibiotic tolerance in. Communications Biology 4.
